# How Can Animal Models Inform the Understanding of Cognitive Inflexibility in Patients with Anorexia Nervosa?

**DOI:** 10.3390/jcm11092594

**Published:** 2022-05-05

**Authors:** Kaixin Huang, Claire J. Foldi

**Affiliations:** 1 Department of Physiology, Monash University, Clayton 3800, Australia; khua0024@student.monash.edu; 2 Monash Biomedicine Discovery Institute, Clayton 3800, Australia

**Keywords:** anorexia nervosa, activity-based anorexia, cognitive flexibility, animal models, prefrontal cortex, serotonin, dopamine

## Abstract

Deficits in cognitive flexibility are consistently seen in patients with anorexia nervosa (AN). This type of cognitive impairment is thought to be associated with the persistence of AN because it leads to deeply ingrained patterns of thought and behaviour that are highly resistant to change. Neurobiological drivers of cognitive inflexibility have some commonalities with the abnormal brain functional outcomes described in patients with AN, including disrupted prefrontal cortical function, and dysregulated dopamine and serotonin neurotransmitter systems. The activity-based anorexia (ABA) model recapitulates the key features of AN in human patients, including rapid weight loss caused by self-starvation and hyperactivity, supporting its application in investigating the cognitive and neurobiological causes of pathological weight loss. The aim of this review is to describe the relationship between AN, neural function and cognitive flexibility in human patients, and to highlight how new techniques in behavioural neuroscience can improve the utility of animal models of AN to inform the development of novel therapeutics.

## 1. Introduction

Anorexia nervosa (AN) is a life-threatening psychiatric disorder that most frequently occurs in young females. It is characterised by significantly low body weight caused by an intense fear of weight gain and relentless behaviours that prevent weight gain, including extreme caloric restriction and excessive exercise [1]. Whereas some patients can recover from AN with or without relapse, symptoms persist in about 20% of patients for more than 10 years and become chronic [2].

Although AN has high rates of relapse, chronicity, and one of the highest rates of mortality among psychiatric disorders, no specific medication is effective for treating AN [3,4,5,6,7]. Whereas there are clearly psychological and social influences involved in the development of AN, it is now well established that AN has underlying genetic and neurobiological causes [1,8]. There are no specific genes known to increase the risk of developing AN; however, a recent genome-wide association study (GWAS) identified eight risk loci that were significantly associated with the condition [9]. Although GWAS findings are informative, identifying strong hypotheses about the role of these risk loci in the development of AN is not straightforward. It is known that the genes identified by GWAS to be associated with AN are significantly correlated with both metabolic and psychiatric traits, including those associated with disorders such as obsessive-compulsive disorder (OCD), depression and anxiety [9]. Still, part of the reason that therapeutic options for treating AN are limited is an inadequate understanding of the underlying neurobiological causes of AN [5]. Current treatments for AN mainly focus on weight outcomes and comorbidities such as OCD, depression and anxiety [1,5,6,7]. Although these strategies may promote weight gain in the immediate term, they are ineffective at providing long-term therapeutic outcomes because they do not treat the core causes of AN [5,7].

### Cognitive Inflexibility: A Characteristic of AN

The inability of patients to change deeply ingrained patterns of thought and behaviour is a key contributor to the persistence of AN [10,11]. This profound genetic and biologically driven cognitive inflexibility prevents adherence to treatment and is the most consistently reported impairment in cognitive function in patients with AN [1,10,11,12]. Broadly speaking, cognitive flexibility enables organisms to modify behaviours according to changing environmental demands [10,12]. In patients with AN, impaired cognitive flexibility is most evident in the rigid adherence to diet and exercise regimes even when faced with rapidly declining body weight [1,10]. In comparison to healthy individuals, patients with AN have poorer performance on neurocognitive tests for flexibility, such as the Wisconsin Card Sorting Task (WCST), the Trail Making Test (TMT) and the Brixton Spatial Anticipation Test [11,13,14,15,16,17]. These impairments also persist after weight regain and even after long-term recovery, suggesting that cognitive inflexibility does not depend on body weight status or the duration of disease [14,15,16]. Furthermore, unaffected sisters and mothers of patients with AN are also cognitively inflexible, indicating that a genetic predisposition to cognitive inflexibility could lead to the development and maintenance of anorectic behaviours in vulnerable individuals [14,18,19]. Moreover, enhanced cognitive flexibility is shown to mediate improvements in daily life functioning, anorectic symptoms and depressive symptoms in patients with AN following extended inpatient treatment [20]. Taken together, these findings suggest that therapeutic strategies targeting cognitive flexibility could be effective at alleviating the core symptoms of AN. Increasingly, psychotherapeutic approaches such as cognitive-behaviour therapy (CBT) and MANTRA (the Maudsley Anorexia Nervosa Treatment for Adults) are being employed in the treatment of AN that specifically address inflexible thinking and behaviour [21,22].

What remains to be determined is whether cognitive inflexibility itself predisposes individuals to develop AN or could be used as a diagnostic tool to predict disease progression and tailor treatment. The elucidation of the direct relationship between cognitive inflexibility and vulnerability of AN may contribute to the development of therapeutic interventions that are effective for AN. These interventions could even be implemented before pathological weight loss becomes too severe and protracted when recovery becomes less likely [15,20,23]. The aim of this review is to present evidence supporting the presence of this relationship and suggest that a better understanding of the cognitive drivers of AN could inform the development of novel therapeutic strategies. We highlight the utility of well-recognised animal models to uncover the neurobiological substrates of cognitive deficits that drive pathological weight loss.

## 2. Cognitive Flexibility: An Essential Executive Function

Along with working memory, inhibitory control and attention, cognitive flexibility is one of the core executive functions, which is largely regulated by the prefrontal cortex (PFC) [12,24]. It is crucial for all aspects of life, allowing a person to identify the changing demands in their internal and external environments, adjust cognitive strategies and efficiently adapt behaviours to respond to the new demands [12,24]. This ability is goal-directed and influenced by reward prospects [12,25,26]. Enhanced cognitive flexibility can contribute to better academic performance [27,28], better creativity [29] and better late-stage quality of life [30]. In contrast, repetitive, rigid thoughts and behaviours are features of cognitive inflexibility, leading to the persistence of inappropriate responses to changing demands even when the behavioural outcome is negative [10,25]. Cognitive inflexibility has been reported in various psychiatric disorders, including OCD, substance abuse, gambling addictions, Major Depressive Disorder (MDD), schizophrenia and bipolar disorder, suggesting that cognitive flexibility is critical to many aspects of mental health [24,25,31,32,33,34,35].

### 2.1. Testing Cognitive Flexibility in Humans

Set-shifting and reversal learning are two aspects of cognitive flexibility [36,37]. In the clinic, these aspects can be assessed by a range of neurocognitive tests. Set-shifting tasks require participants to shift attention from one characteristic of the stimulus to another, while reversal learning tasks require participants to inhibit and modify recently acquired behaviours in response to changed rules or stimulus–reward associations [36,37].

**Figure 1:**
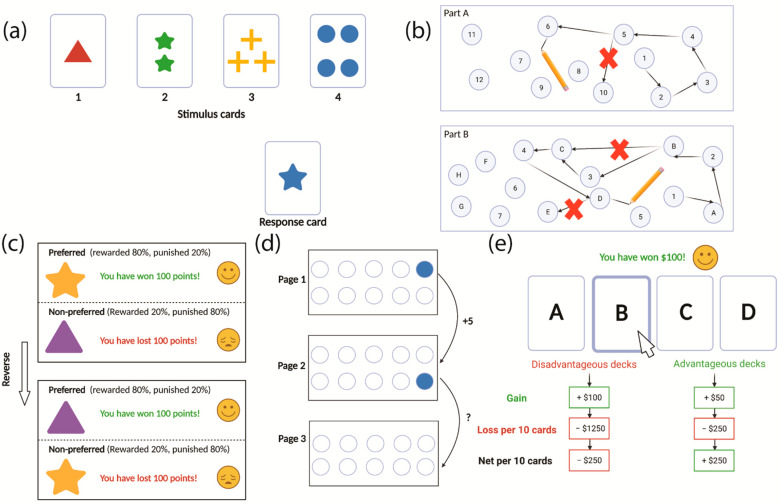
Common cognitive tests that are used to assess cognitive flexibility in humans. (**a**) The Wisconsin Card Sorting Task (WCST) is the most commonly used test in the examination of cognitive flexibility. Participants need to match the response card to the stimulus cards based on either colour, shape or number. The sorting rule changes after several consecutive correct matches without warning. (**b**) The Trail Making Test (TMT) is used to assess set-shifting. Participants need to link the circled numbers in sequence in part A, while linking alternating numbers and letters in order in part B. (**c**) The probabilistic reversal learning task is used to assess reversal learning. Participants need to learn that the two stimuli are associated with different levels of reward and generate preference for the stimulus with high reward. The reward–stimulus association will be reversed without warning once they learn the relationship. (**d**) The Brixton Spatial Anticipation Test focuses on reversal learning. Participants need to predict the position of the filled circle on the following page according to the rule learnt from the previous page. (**e**) The Iowa Gambling Task (IGT) is used to assess decision-making and flexibility. Participants are required to maximise profit by preferentially choosing cards from the advantageous over the disadvantageous card decks.

The Wisconsin Card Sorting Task (WCST) is the most widely used task in the assessment of set-shifting [10]. In the WCST, four stimulus cards showing stimuli that are different in shape, number and colour are presented to the participants. A response card is given to the participant, and they are required to match it to one of the stimulus cards according to either shape, colour or number without a given sorting rule. Feedback on whether the matching is correct is then given by the experimenter after each response. The participant is expected to learn the sorting rule according to the feedback given. After several consecutive correct responses, the sorting rule is changed without informing the participant. As such, they are required to learn the new sorting rule to generate correct responses [38] (Figure 1a). To respond correctly, the participant needs to shift attention from one characteristic of the stimulus card (such as colour) to another (such as number). Failing to flexibly modify cognitive strategies in this task results in perseverative errors, in which the participant applies a sorting rule despite receiving negative feedback. Thus, a high number or proportion of perseverative errors in this task indicates impaired cognitive flexibility [38]. The WCST has been used in numerous studies to demonstrate deficits in set-shifting [11,13,16,19]. However, it has also been argued that this task is not specific to set-shifting, but also reflects a level of reversal learning, because the participant is required to identify and adjust their behaviour in response to the changed sorting rule according to feedback [36].

Other tasks used to assess cognitive flexibility include the Trail Making Test (TMT), probabilistic reversal learning task, the Brixton Spatial Anticipation test and the Iowa Gambling Task (IGT). The TMT is used to assess set-shifting [39]. It requires the participant to first connect the randomly placed numbers in numerical sequence (Part A), and then draw a line to link alternating numbers and letters in order (Part B; Figure 1b). The time taken to complete each part is recorded. When an error occurs, the experimenter points it out immediately, which could extend the time taken to finish the task. In this task, Part A provides a baseline measurement of motor speed while the additional response time taken in Part B reflects set-shifting ability. Hence, a large difference between the time taken in Part A and Part B indicates cognitive inflexibility [39]. A probabilistic learning task can be used to measure reversal learning in humans, which involves a serial selection of “preferred” versus “non-preferred” stimuli that are informed by feedback provided after each response. The “preferred” stimulus is rewarded in 80% of the trials, whereas the other is rewarded in only 20% of the trials. After a number of trials, the reward–stimulus association is reversed without warning. The participant is required to adjust their choice behaviour in order to retain the high reward [35] (Figure 1c). Reversal learning can also be measured without “rewards” per se using the Brixton Spatial Anticipation Test, in which rules pertaining to the correct spatial arrangement of numbers and filled circles are learned and then changed without warning, in a similar fashion to the probabilistic reversal learning task described above [20] (Figure 1d). A high number of incorrect responses in this task indicates that the participant is not able to flexibly modify cognitive strategies [40]. Moreover, the IGT can be used to assess flexible decision-making in which the participant is required to make choices between immediate reward and delayed punishment. In this task, the participant selects a card from one of the four card decks. Two of these decks are designated “high reward” (e.g., Decks A and B), and a choice from these results in a large amount of money. The other two decks are designated “low reward” (e.g., Decks C and D), and a choice from these results in a small monetary reward. However, the punishment (or “loss”) associated with the “high reward” card decks is higher than that associated with the “low reward” decks, and repeated selection from the ‘high reward” decks will result in a net loss after 10 consecutive choices. Hence, Decks A and B are considered disadvantageous, while Decks C and D are advantageous in the long-term. Thus, failing to increase advantageous choices over time in this task reflects deficits in decision-making [41].

### 2.2. Testing Cognitive Flexibility in Rodents 

Due to technical limitations, ethical considerations and the complex nature of the human brain, it is challenging to study the neuronal mechanisms underlying cognitive processes in humans [42,43]. Whereas the overall involvement of a particular brain region or pathway can be revealed in human subjects, the functional interactions between different pathways are often unresolvable [44,45]. Hence, animal models are critical in elucidating the neuronal correlates of cognition and the underlying mechanisms of cognitive dysfunctions in human psychiatric disorders [42]. Rodent models, in which neuronal activity can be easily and precisely manipulated and recorded using advanced techniques, have been widely used in the investigation of brain–behaviour relationships [42,46]. Techniques such as optogenetics have been combined with cognitive tasks to reveal the role of specific brain regions in cognitive flexibility in rodents [47].

**Figure 2:**
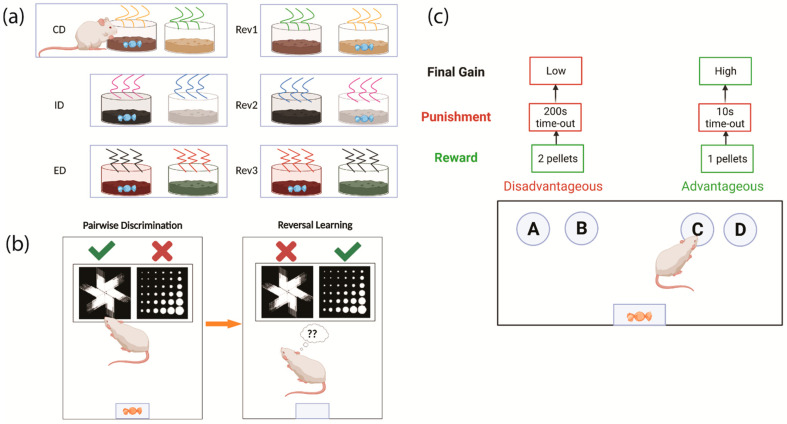
Cognitive tests for assessing cognitive flexibility in rodents. (**a**) The attentional set-shifting test (ASST) assesses both set-shifting and reversal learning. In the compound discrimination (CD), intradimensional shift (ID) and extradimensional shift (ED) stages, the rodent needs to switch attention either between two odours or between odour and digging medium to identify the reward-related stimulus. The ID and ED phases are thus used to assess set-shifting. The reward-stimulus association is changed in the reversal stages (Rev1,2,3) between the set-shifting stages (ID and ED) to assess reversal learning. (**b**) Reversal learning tasks can also be performed on a touchscreen. In the task, the rodents first learn to discriminate between the two visual stimuli and identify the one associated with reward. Once the reward-stimulus association is acquired by the rodent, it will be reversed, and the rodent needs to learn that the previously rewarded stimulus has become unrewarded and vice versa. (**c**) The Iowa gambling task (IGT) can be also used in rodents. In this task, rodents are required to choose between four nose-poke holes, varying in associated rewards and punishments. The advantageous choices are associated with less immediate rewards but short punishment time, whereas disadvantageous choices are associated with more immediate rewards but longer punishment time. CD: compound discrimination; Rev 1: first reversal; ID: intradimensional shift; Rev 2: second reversal; ED: extradimensional shift; Rev 3: third reversal.

The attentional set-shifting test (ASST) is a common method used to examine cognitive flexibility in rodents. It is adapted from a modified version of the WCST that allows the independent measurement of set-shifting and reversal learning [48,49]. The standard ASST includes seven stages, in which the rodent is first required to identify the rewarded stimulus from a pair of stimuli in the same dimension (such as odour). Once the rodent reaches a certain number of consecutive correct trials in the first simple discrimination stage, they progress to the compound discrimination (CD) stage. A pair of stimuli in the other dimension (in this case, digging medium) is introduced in this stage, while the rewarded odour from the first stage remains unchanged. Then, the rodent is required to perform the first reversal (Rev1), in which the reward–stimulus association is reversed. The next stage is the intradimensional (ID) shift involving the discrimination of the reward-related odour between the new pair of odours. The ID stage is followed by the second reversal (Rev2). The extradimensional (ED) stage is the sixth stage, where the rodent is required to ignore the odours and identify the rewarded stimulus from the pair of different digging mediums. The last stage of ASST involves a reversal task (Rev3), in which the rewarded digging medium in the ED stage is now unrewarded (Figure 2a). The number of trials needed for the rodent to meet the criterion in every stage is recorded. A high number of trials in ID and ED indicates poor set-shifting, whereas more trials needed in the three reversal stages reflects impaired reversal learning [37,49,50].

Reversal learning tasks for rodents can also be performed using a touchscreen platform, which has practical similarities to human cognitive tests and therefore possibly greater translational relevance [51]. Rodents are required to discriminate a rewarded stimulus from a pair of visual stimuli presented on the touchscreen. When this discrimination is completed with over 80% accuracy, the reward–stimulus association is reversed in a similar fashion to the probabilistic reversal learning task in humans. The number of errors made in each session and the trials needed to meet the accuracy criterion are used in the analysis of performance [52] (Figure 2b). The IGT has also been modified and used in rodents. In a similar fashion, rodents are required to make decisions between four nose-poke holes. Choices A and B are disadvantageous, resulting in an immediate reward of two food pellets, followed by longer time-outs as penalty, whereas Choices C and D, delivering only one food reward but with shorter penalty times, are advantageous. The percentage of advantageous choices in the last 20 min of the 1-hour test reflects the ability of rodents to make economic decisions in the long term [53].

### 2.3. Neuronal Control of Cognitive Flexibility

Damage to parts of the PFC is shown to worsen performance on set-shifting and reversal learning tasks in humans, non-human primates and rodents, indicating that the PFC plays a key role in cognitive flexibility [47,50,54,55,56,57,58,59]. In humans, the PFC can be broadly divided into the medial prefrontal cortex (mPFC), anterior cingulate cortex (ACC), lateral prefrontal cortex (lPFC) and orbital frontal cortex (OFC) based on their differential functions and anatomical locations [60,61]. Although human and rodent brains are structurally different, the mPFC of rodents is functionally analogous to the dorsolateral prefrontal cortex (dlPFC) and ACC in humans and non-human primates [62]. These subregions, particularly the mPFC and OFC, have critical roles in mediating cognitive flexibility [61,63]. In addition, the neurochemical circuits that employ dopamine (DA) and serotonin (5-HT) as neurotransmitters have widespread interactions within the mPFC and subcortical regions, and are also involved in the mediation of cognitive flexibility [63]. 

One particular pathway that seems to be critical for cognitive flexibility extends from the mPFC to the striatum [64,65,66], and contributes to reward-related behaviours through its actions on pathways between the ventral tegmental area (VTA) and striatum [67]. Since both PFC and striatum can receive DA input from the VTA, DA might also have a role in regulating cognitive flexibility [63,66]. In humans, reduced DA levels can alter these frontostriatal pathways and result in poor performance in the WCST [68]. In animal studies, altered dopamine signalling seems to have differential effects on cognitive flexibility depending on the brain region or projection pathway examined. For example, depletion of DA in the dorsomedial striatum and the ventral striatum, particularly the nucleus accumbens (NAc), is associated with impaired reversal learning [66,69,70]. However, hyperdopaminergic states, induced systemically by cocaine or D-amphetamine or via chemogenetic stimulation of VTA-NAc projection neurons, can also impair cognitive flexibility in a reversal learning task [71]. Moreover, blocking the DA receptors expressed in the mPFC, D1 and D2, caused impairment in set-shifting [36,72]. Notably, the depletion of DA in the OFC did not affect reversal learning in spite of the key role of OFC in this aspect of cognition [73]. Whereas DA in the PFC primarily regulates set-shifting and reward-related behaviours, 5-HT acts on neurons in the PFC to control reversal learning and punishment-related behaviours [10]. In non-human primates, the depletion of 5-HT in PFC worsens performance in reversal learning without affecting set-shifting, suggesting that the effect on 5-HT is specific to reversal learning [73,74]. This effect of 5-HT depletion on reversal learning has also been observed in rodents, in which impaired reversal learning is associated with localised 5-HT depletion in the OFC but not mPFC [75]. Collectively, these studies elucidate the predominant role of the mPFC and DA systems in regulating set-shifting and reward-related behaviours, as well as the contributions of the OFC and 5-HT systems to the control of reversal learning and punishment-related behaviours.

## 3. Common Neurological Drivers of AN and Cognitive Flexibility

Although the current understanding of the pathophysiology of AN is limited, the utility of brain imaging techniques, such as positron emission tomography (PET) and functional magnetic resonance imaging (fMRI), has provided some insights into the neurobiology underpinning AN [8,44,76]. For example, anhedonia, altered interoceptive awareness and feeding behaviours observed in patients with AN are associated with disturbances in PFC, striatum, amygdala, insular cortex and hypothalamus, as well as the DA and 5-HT neurotransmitter systems [8,76]. These findings imply that AN and cognitive inflexibility might share the same neurological drivers, including disturbed functions in the PFC, as well as the DA and 5-HT neurotransmitter systems [76]. 

### 3.1. Neurobiology Underlying AN

Although hypothalamic dysfunctions are responsible for the deficient regulation of food intake and energy homeostasis in AN, negative energy balance is signalled to the rest of the brain via the hypothalamus which is a driver of state-dependent maladaptive behaviour [44,76,77,78]. The recent focus of AN has therefore shifted to these wider brain circuits that control aspects of behaviour contributing to state-independent drivers of pathological weight loss [44,76]. One major focus is on the imbalance between control and reward neurocircuits. Specifically, cognitive control is often exaggerated in AN, while reward processing is diminished [8,44,45]. These activities are governed by projections between the PFC and the striatum known as “corticostriatal circuits”. Within these pathways, the PFC is responsible for cognitive control, whereas the striatum regulates reward processing [8,44]. In addition, the DA and 5-HT neurotransmitter systems, which regulate reward and inhibitory control, are also widespread within these regions [8].

Individuals with AN commonly demonstrate excessive self-control in response to food-related stimuli that persists after weight recovery [79,80,81,82]. Excessive self-control in food choice is associated with enhanced dlPFC activity [80], which is exaggerated both in patients who are currently ill with AN (acute phase of AN) and weight-recovered from AN, and leads to attenuated reward processing and the avoidance of food [83,84]. This aligns with a finding that women who recovered from AN show decreased activity in striatum in response to the taste of pleasant food [85].

**Figure 3:**
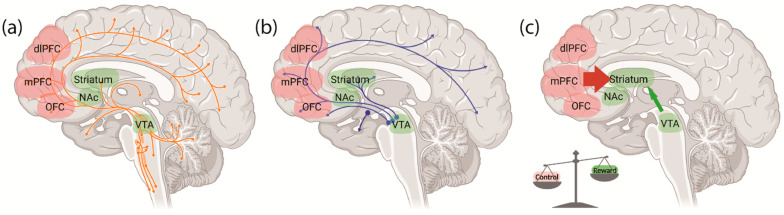
Neural circuits and brain areas involved in AN. (**a**) The 5-HT and (**b**) DA circuits are shown to be disrupted in patients with AN, contributing to an (**c**) imbalance between the reward and control neurocircuits in AN. The activity of prefrontal regions that are responsible for cognitive control is enhanced, while the subcortical regions that regulate reward processing are hypoactive in individuals with AN. dlPFC: dorsolateral prefrontal cortex; mPFC: medial prefrontal cortex; NAc: nucleus accumbens, OFC: orbitofrontal cortex; VTA: ventral tegmental area.

Although the use of food-related stimuli provides direct insight into food-related reward processing in AN, it is possible that the diminished reward processing is caused by an insensitivity to food-related rewards rather than the effect of excessive cognitive control over reward processing, considering that patients with AN may not consider food as a positive reinforcer [45,86,87]. Thus, monetary rewards are used in many studies investigating reward processing in AN. They often reveal that exaggerated self-control in patients with AN is not limited to food [88], but that individuals recovered from AN also show elevated activation in cognitive control circuits when making decisions on the value of monetary rewards [89] and fail to inhibit the activity of dlPFC when receiving positive feedback [90]. Taken together, these studies are consistent with the studies which have used food-related stimuli, confirming that PFC activity is exaggerated in AN, leading to excessive cognitive control and diminished reward processing (Figure 3).

Altered DA and 5-HT signalling could contribute to the imbalance between reward and control in patients with AN, considering the roles of these transmitter systems in motivation, mood and feeding behaviour [44,91,92,93]. Individuals recovered from AN show reduced DA metabolism [92,94] and elevated D2/3 receptor expression, which reduces activation of the DA system [95,96] and is correlated with harm avoidance in patients with AN [95,96]. Interestingly, computational modelling of choice behaviour in the IGT revealed that patients with AN demonstrate reduced loss aversion, which may play a role in the insensitivity of patients to the negative consequences of the illness itself [41] and is known to be mediated by dopamine [97]. Moreover, AN is associated with increased 5-HT metabolism [98], and an inappropriate balance of 5-HT_2A_ and 5-HT_1A_ receptor expression in cortical regions [99,100]. Overall, the disturbances in DA and 5-HT neurotransmitter systems could also contribute to the imbalance between inhibition and reward in AN. It should be noted that human studies reporting changes in DA and 5-HT signalling in AN have limited resolution due to the complex and diverse nature of transmitter systems that have different functional outcomes depending on their inputs and projections. This caveat further supports the utility of animal models for understanding the specific roles of transmitters such as DA and 5-HT in pathological weight loss and cognitive inflexibility. In animal models, these systems can be perturbed in a region- and receptor-specific manner to reveal the detailed mechanisms underlying their associations.

### 3.2. Cognitive Flexibility in AN

Given the overlapping neurobiological drivers of AN and cognitive inflexibility, it is not surprising that cognitive inflexibility is consistently reported in individuals with AN. For example, poor performance on the WCST has been demonstrated in adolescents and adults with AN, patients recovered from AN, as well as the unaffected sisters and mothers of AN patients [11,13,14,16,17,18,19,101]. Although the participants who have recovered from AN appear to have fewer preservative errors than those who are currently suffering from AN, impaired cognitive flexibility persists after long-term recovery [15,16]. Moreover, the finding that unaffected sisters and mothers of patients with AN also exhibit cognitive inflexibility highlights an underlying genetic contribution [18,19]. Indeed, genetic overlaps have been found between AN and OCD, which is a psychiatric disorder characterised by rigid and compulsive behaviours [9,102]. OCD is also considered a risk factor for AN [1,102]. Neuroimaging in patients with AN during performance on cognitive tasks has shown the involvement of various cortical subregions that are differentially activated during flexible choice behaviour [10,11,103].

Although rigidity appears to be an important feature in patients with AN, these reports are not entirely consistent considering that cognitive flexibility was not impaired in patients with AN in some studies using WCST, TMT and other cognitive tasks, such as verbal flexibility tasks [104,105,106]. The discrepancies in demographics of participants and methods of cognitive testing used in the studies may contribute to the inconsistent findings [104]. For example, while no significant differences in WSCT performance were found between adolescents with AN and healthy controls in one study, the duration and severity of AN were not reported [105]. In addition, although patients with AN demonstrated worse performance in the TMT and Brixton test, no significant differences were shown in the performance of the verbal flexibility task, indicating discrepancies in different domains of cognitive flexibility [106]. To understand why these reports are not consistent, the relationship between cognitive flexibility and AN must be investigated in greater detail, including the direct and causal associations between altered neuronal function and cognition relevant to AN, which can only be achieved using animal models.

## 4. The Utility of the Activity-Based Anorexia (ABA) Model for Understanding the Neurobiology of AN

The activity-based anorexia (ABA) model is the most widely used animal model of AN, which mimics features of AN, including rapid body weight loss, self-starvation and hyperactivity [107,108]. Similar to the human condition, young rodents are more vulnerable to ABA, and susceptibility to ABA is influenced by early-life experience [107,109,110,111,112]. In addition, the expression of neuronal cell adhesion molecule 1 (NCAM1), which is elevated in ABA mice in response to food restriction, was found to be associated with a risk multigenic locus on chromosome 3 in patients with AN [9].

In the ABA paradigm, time-restricted food access is combined with unlimited access to a running wheel, leading to reduced food intake, elevated activity levels and hence, extreme weight loss [107,108]. Weight loss in the ABA model is thought to be a result of failure in adapting to the experimental feeding schedule [113]. Although hyperactivity during food restriction increases weight loss in ABA animals [114,115,116], increased activity prior to scheduled food access, which is known as food anticipatory activity (FAA), is associated with reduced body weight loss in ABA [116,117]. FAA is a motivated behaviour that only develops when animals are on a time-fixed feeding schedule [113,118]. It is regulated by dopaminergic reward circuits and hypothalamic homeostatic circuits, which are also involved in modulating food intake [114,118]. Indeed, increased FAA has been found to be linked with increased food intake, which is also a protective factor against weight loss in ABA [116,117]. In contrast, factors including increased physical activity, low initial body weight, low food intake and social isolation contribute to susceptibility to ABA [109,115,119].

Interestingly, in contrast to the fact that AN is more common in women, inconsistent results have been found in sex differences in susceptibility to the ABA model [1,120,121]. While either no sex differences [109], or greater susceptibility in females [111] or in males [122] was observed in rats, greater susceptibility to ABA was demonstrated in male C57BL/6 mice [120]. Whereas biological factors, such as reproductive hormones, and psychosocial factors, such as cultural and social pressures rewarding “thinness”, may account for the higher prevalence of AN in women, sex differences in body fat composition, behaviours and hormonal levels in response to stress and elevated energy expenditure may account for the sex differences in susceptibility to ABA in rodents [120,121,122]. However, the underlying mechanisms remain to be elucidated [120].

Consistent with the human condition, disrupted 5-HT systems also play a role in ABA [44,108], with the direction and behavioural specificity of these effects being equally complicated in the rodent model. For example, fluoxetine, which increases circulating 5-HT by inhibiting reuptake, attenuates hyperactivity and increases food intake in ABA mice without increasing survival [123], whereas fenfluramine, which promotes 5-HT release, reduces food intake and accelerates weight loss in ABA rats [124]. Moreover, physical activity and weight loss in ABA rats is diminished following the suppression of 5-HT activity with 8-OH-DPAT; however, in this case, food intake was not affected [125]. The inconsistent findings in the effect of 5-HT on food intake could be attributed to the complex nature of the 5-HT system, which includes multiple subtypes of receptors and functional outcomes that vary in different brain regions. The effect of altered 5-HT systems on food intake in ABA could therefore be hindered by the non-selective manipulation of the systems that occurs with systemic administration of serotonergic compounds [44]. Furthermore, the effects of 5-HT on feeding behaviours could also be dependent on the subtypes of 5-HT receptor activated, which have differential effects on reward and food intake [126].

DA systems also play a critical role in the ABA phenotype in rodents, with the selective blockade of D2/3 receptors being protective against weight loss during ABA [96,127]. In addition, olanzapine, which is an antagonist of both DA receptors and 5-HT receptors, increases survival in ABA mice via suppressing FAA, suggesting an underlying role of DA receptors in regulating weight loss in ABA [123]. Moreover, a DA transporter (DAT) knockout model revealed both excessive DA activity in striatal regions, and accelerated weight loss and hyperactivity during ABA, suggesting that excessive DA could contribute to hyperactivity and greater vulnerability to ABA [110]. However, while feeding behaviour during ABA is associated with increased DA release in NAc, this elevation was not observed during FAA, indicating a disconnect between DA and motivated activity [128]. Together, these findings suggest that the role of pathway-specific DA in the development of ABA might be different from global DA. Indeed, our previous work demonstrated that activation of the specific pathway extending from VTA to NAc in rats increased food intake during ABA and thus prevented weight loss [114], revealing a direct role of activity with this primarily dopaminergic pathway on the development of and rescue from ABA.

## 5. Investigating Cognitive Inflexibility in ABA

The ABA model has also shown parallel links with the human condition with respect to cognitive inflexibility. Performance on the ASST has been examined in female rats before and after exposure to ABA, as well as after recovery from ABA. Compared to a pair-fed control group, rats exposed to ABA conditions required significantly more trials to reach criterion in the reversal phases of ASST, indicating that ABA had a deleterious effect on reversal learning. However, no significant difference between groups was found in the performance in the SD, CD, ID and ED phases, suggesting ABA had no effect on discrimination learning or set-shifting [37]. Although the finding of ABA-induced impaired reversal learning is in line with human studies, the finding of unaffected set-shifting is not [13,14,15,16]. In addition, impaired reversal learning was no longer observed in the ABA group after weight recovery [37], unlike the persistence of cognitive inflexibility in humans after recovery from AN [14,15,16]. Thus, ABA may be more suitable for investigating the early phase of AN, as the effect of long-term starvation is not investigated in the ABA model [37]. Moreover, spatial cognition has been shown to improve after weight restoration in ABA rats, indicating that there are specific alterations to different cognitive domains that are influenced by restricted feeding and weight loss [129]. Since AN is a chronic disease, efforts have been made to establish a modified chronic ABA model in which animals experience starvation and low body weight for over 15 days [130]. However, as food access is amount-restricted in this chronic model, the critical feature of the standard time-restricted ABA model that requires animals to choose between food and the running wheel, and learn to adapt to the feeding schedule, is lost. Thus, it may be less suitable for the investigation of cognitive flexibility [93]. Despite the limitation of the short-term starvation, the concept of pathological weight loss developing via a maladaptive and persistent choice to run instead of eat highlights that PFC function is likely to be altered in the standard ABA model [37]. 

Indeed, recent work from our laboratory demonstrated that suppression of activity within a corticostriatal circuit extending from neurons in the mPFC to the nucleus accumbens shell (AcbSh), which is a part of the ventral striatum, completely prevented weight loss associated with exposure to ABA conditions [131]. Furthermore, suppression of this same pathway in a separate cohort of rats improved cognitive flexibility in a touchscreen-based reversal learning task. In contrast, activation of this corticostriatal circuit both worsened body weight maintenance in ABA and increased perseverative errors in the reversal learning task [131]. This study provided the first direct evidence of a neurobiological link between cognitive flexibility and pathological weight loss, and supports the use of the ABA model in elucidating the direct role of cognitive flexibility in AN [131]. However, due to the extensive testing time required to examine reversal learning using the touchscreen-based task employed in this study, it was impossible to test cognitive flexibility and susceptibility to weight loss in ABA in the same animals in order to identify a causal relationship between the two. Therefore, new approaches to assess cognitive flexibility in rodents are required. Radio-frequency identification (RFID) technology, which enables automated animal sorting and high-throughput testing, offers a potential solution [132,133].

## 6. New Technologies to Improve the Assessment of Cognition and Behaviour in Animal Models of AN

The use of touchscreen-based operant testing chambers increases the translational relevance of cognitive testing in rodents as these are designed to match the frequently used touchscreen-based testing systems in humans. These are based on the Cambridge Neuropsychological Test Automated Battery (CANTAB), which is a validated set of computerised tests that assesses different aspects of cognitive function such as attention and working memory in humans [52,134,135]. By combining the touchscreen operant platform for rodents with RFID-based tracking, it is now possible to conduct highly standardised and translationally relevant cognitive testing with minimal human intervention [52,132,134]. This is particularly important considering that human intervention itself can cause behavioural alterations in rodents, inducing confounding factors to the experimental outcomes of cognitive tasks [136]. For example, the handling of rodents, which is required when transferring the animal to the testing chamber in the conventional touchscreen tests, can affect attention and emotion, disrupt behaviours and, importantly, influence susceptibility to ABA in adolescent rats [112,136,137]. In addition to the elimination of human intervention, automation of access to touchscreen chambers allows animals to test themselves in a “self-directed” manner and engage in task acquisition at any time of the day or night, facilitating rapid testing with substantially reduced experimental labour [132]. Incorporating such a system for examination of cognitive flexibility in ABA rats will allow the direct identification of the cognitive predictors of susceptibility to pathological weight loss, which is thus far based largely on post hoc correlation studies in clinical populations.

## 7. Conclusions

Cognitive inflexibility has been considered as a trait marker of AN that contributes to the development of the disorder and persistence of symptoms [10]. This review highlights that cognitive inflexibility and AN share common neurological drivers, including disrupted prefrontal circuitry, and dysregulated DA and 5-HT signalling in specific brain regions and pathways. The evidence suggests that novel pharmacotherapeutic strategies targeted at modulating prefrontal serotonin and/or striatal dopamine function may be effective in treating the cognitive inflexibility that characterises AN. Whereas evidence from animal studies identifies specific PFC projections that are involved in both pathological weight loss and cognitive flexibility, the direct relationship of this association has not yet been identified. The ability to study complex cognitive tasks in rodent models rapidly, without experimenter intervention, will allow this direct relationship to be tested. Moreover, whether cognitive inflexibility predisposes individuals to developing AN is not clear. Animal studies that incorporate tests of flexible learning and well-established means with which to elicit anorectic symptoms in the same animals improves the utility of animal models, such as ABA, for elucidating these types of predisposing factors. Future studies using experimenter free and high-throughput cognitive testing paradigms with translational relevance will shed light on the specific neurobiological underpinnings of cognitive dysfunction in ABA in order to inform the development of effective medicinal therapeutics for AN.
